# Adaptive Artifact Removal From Intracortical Channels for Accurate Decoding of a Force Signal in Freely Moving Rats

**DOI:** 10.3389/fnins.2019.00350

**Published:** 2019-04-16

**Authors:** Abed Khorasani, Vahid Shalchyan, Mohammad Reza Daliri

**Affiliations:** ^1^Neuroscience and Neuroengineering Research Lab, Department of Biomedical Engineering, School of Electrical Engineering, Iran University of Science and Technology (IUST), Tehran, Iran; ^2^Kerman Neuroscience Research Center, Institute of Neuropharmacology, Kerman University of Medical Sciences, Kerman, Iran

**Keywords:** brain–machine interface, artifact removal, adaptive common average reference filter, adaptive filtering, Kalman filter, neural decoding

## Abstract

Intracortical data recorded with multi-electrode arrays provide rich information about kinematic and kinetic states of movement in the brain–machine interface (BMI) systems. Direct estimation of kinetic information such as the force from cortical data has the same importance as kinematic information to make a functional BMI system. Various types of the information including single unit activity (SUA), multiunit activity (MUA) and local field potential (LFP) can be used as an input information to extract motor commands for control of the external devices in BMI. Here we combine LFP and MUA information to improve decoding accuracy of the force signal from the multi-channel intracortical data of freely moving rats. We suggest a weighted common average referencing (CAR) algorithm in order to valid interpretation of the force decoding from different data types. The proposed spatial filter adaptively identifies contribution of the common noise on the channels employing Kalman filter method. We evaluated the efficacy of the proposed artifact algorithm on both simulation and real data. In the simulation study, the average *R*^2^ between the original and reconstructed signal of all channels after applying the proposed artifact removal method was computed for input SNRs in the range of −45 to 0 dB. Weighted CAR method can effectively reconstruct the original signal with average *R*^2^ higher than 0.5 for input SNRs higher than −s10 dB in case of adding simulated outlier and motion artifacts. We also show that the proposed artifact removal algorithm 33% improves the accuracy of force decoding in terms of *R*^2^ value compared to standard CAR filters.

## Introduction

Brain–machine interface (BMI) provides an alternative artificial route for transmitting the brain commands to the patient’s limbs with the goal of movement restoration in different neurological disorders such as stroke and spinal cord injury (Hochberg et al., 2012; Collinger et al., 2013; Bouton et al., 2016). Selecting the optimal brain inputs to decode motor commands with high longevity and accuracy is an unsolved question among neuroscientists. The multiunit activity (MUA) defined as un-sorted single unit activities provides a high-resolution movement related information with greater stability than single unit activities (SUA) (Chestek et al., 2011). Furthermore, local field potential (LFP) signals recorded from the motor cortex is represented as more robust and stable signal in neural interface devices (Flint et al., 2012). Recent studies show that different kinematic parameters such as position and velocity can be decoded from the MUA and LFP as precisely as spiking activities (Mehring et al., 2003; Flint et al., 2012, 2013). Furthermore, some studies suggest that combining LFPs and spike information improves the decoding accuracy of these kinematic information (Mehring et al., 2003; Bansal et al., 2012). Mehring et al. (2003) compared decoding performance of arm kinematics from multi-channel LFPs, MUA and SUA. They presented that mixing LFP with MUA lead to more accurate movement decoding accuracy compared with combination of other signal types. Ideal control of an external device based on the BMI techniques depends on accurate decoding of both kinematic such as hand trajectories and kinetic information such as force and torque values. In Khorasani et al. (2016) we showed that force information can be decoded from the low number of LFP channels of motor cortex area. In the current study, we decode continuous force signal from the combination of LFP and MUA signals and compared this decoder with MUA-only and LFP- only decoders. However, recording intracortical signals in the freely moving conditions may affect force decoding accuracy. Various types of artifacts may contaminate intracortical signals in the less constrained conditions. Electrical muscle activities have high frequency characteristics and are produced due to fast muscle activation such as chewing (Shimoda et al., 2012). Furthermore, motion artifacts are often produced due to various factors such as respiration, sudden mechanical pressure on the electrodes or connecting wires and head movements and have the low frequency characteristics (Sweeney et al., 2012). These types of artifacts normally have frequency overlapping with broad-band LFP signals, occur on specific channels and often have higher amplitude than signal of interest. Therefore, using frequency-domain filter or non-automatic spatial filtering cannot effectively remove these complex artifacts (Sweeney et al., 2012).

In this study, we propose an automatic, real-time artifact removal algorithm based on the combination of common average referencing (CAR) filter with Kalman filtering algorithm. We formulate intracortical recordings based on the state-space representation and model the common noise of the brain channels with an autoregressive (AR) structure. In this framework, the weights of mean CAR filter for each channel are updated sample by sample using a Kalman filter algorithm. We first evaluate the functionality of the proposed algorithm in a simulation study with different types of artifacts and different types of signal and noise mixing. Then, we demonstrate the efficiency of this method on removing artifacts from the real multi-channel data recorded from freely moving rats. We show that the proposed method significantly improves the decoding accuracy of a force signal using LFP data or combination of LFP and MUA compared to conventional CAR-based spatial filtering methods.

## Materials and Methods

### Spatial Filtering

In this section, we demonstrate different spatial filters which are used in this study to improve signal quality of intracortical channels. First, we introduce the theory behind two commonly used algorithms in BMIs, mean CAR and median CAR, and explain the main drawback of them to be used for real BMI applications. Then, the proposed weighted CAR filter is explained.

#### Common Average Reference (CAR) Filter

In the CAR filtering, we assume that the observed intracortical signal z_*i*_(*t*) at channel *i* and time *t*, is the mix of a clean brain signal *s_i_*(*t*) and an artifact term *n*(*t*) distributed through the recording channels:

(1) $${\text{z}}_{i}(t)=s_{i}(t)+n(t)$$

where *i* = 1, 2, ..., *K* with *K* total number of channels and *t* = 1, 2, ..., *L* with *L* total number of sample points. In CAR algorithm, noise term can be estimated by computing average of all channels assuming that the common noise was contributed similarly on all channels:

(2) $$\hat{n}(t)\text{=}\frac{1}{K}\sum_{i=1}^{K}{\text{z}}_{i}(t)$$

where $\hat{n}(t)$ shows the average of all channels as estimate of the noise. Therefore, the clean intracortical signal *ŝ_i_*(*t*) corresponding to channel *i* and time *t* can be simply computed by removing average of all channels from each individual channel:

(3) $${\hat{s}}_{i}(t)={\text{z}}_{i}(t)-\hat{n}(t)$$

The main drawback of the CAR filter is that in some situations such as existence of channel-specific noise, the CAR propagates the noise to the clean channels. Furthermore, difference in the amplitude and polarity of the noise in different channels can lead to inaccurate estimation of noise.

#### Median CAR Filter

In median CAR, the median of all channels is used to estimate the noise at each time point:

(4) $$\begin{array}{l} \hat{n}(t)=z{\left(t\right)}_{\left((K+1)/2\right)}\;\;\mathrm{if K is odd}\\ \hat{n}(t)=z{\left(t\right)}_{\left(K/2\right)}+z{\left(t\right)}_{\left(K/2+1\right)})/2\;\;\mathrm{if K is even} \end{array}$$

where *K* denotes the total number of channels. The median is more robust against the outliers in the data compared with mean parameter. However, in normal condition, median CAR may remove the task-related information from the brain data due to its non-linear structure (Rousseeuw and Hubert, 2011).

#### Weighted CAR

**FIGURE 1:**
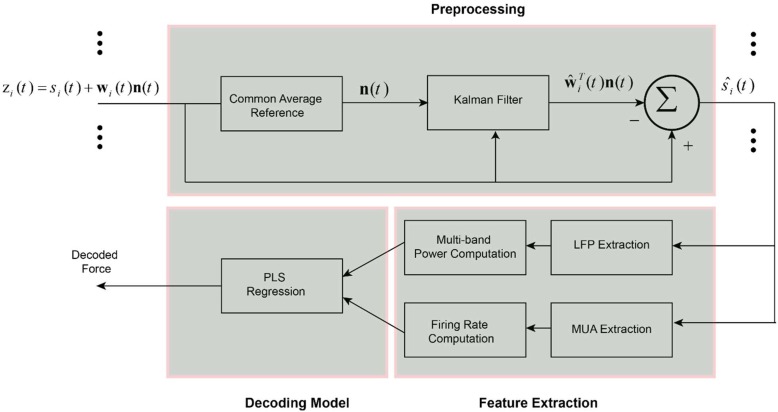
Schematic of the decoding method for predicting force information from the multi-channel intracortical data. In the first stage, the raw brain signals were spatially filtered based on the proposed weighted CAR filter. The weights of common noise were adaptively estimated employing Kalman filter. Different types of information including LFP features, MUA features and the combination of LFP and MUA features were extracted from the pre-processed data in the previous stage. Obtained features were fed into a partial least square (PLS) regression model to create a force decoding model based on the different types of input information.

The main limitation of the standard CAR is that it assumes the common noise has been similarly propagated on the channels. In this study, we model the recoded intracortical signal z_*i*_(*t*) as a combination of a clean signal *s_i_*(*t*) and a noise term *n*(*t*) with autoregressive (AR) structure:

(5) $${\text{z}}_{i}(t)=s_{i}(t)+w_{i}{\left(t\right)}^{T}n(t)$$

where **n**(*t*) = [*n*(*t*) *n*(*t* − 1) ... *n*(*t* − *M* + 1)]^*T*^ is the noise vector containing the temporal values of *n*(*t*) in a time window with *M* values. Also, **w**(*t*) shows weights of the noise term defined as **w**(*t*) = [ *w*_1_ *w*_2_ ... *w_M_*]^*T*^. In this framework we consider that common noise is distributed on the brain channels with different amplitude and polarity. Also, we considered an AR dynamic for the common noise to account the temporal changes of the noise in the channels. We employed Kalman filtering algorithm to adaptively find the true weight vector **w**_*i*_(*t*) for each channel *i*. The schematic representation of the proposed spatial filter is shown in Figure 1. To estimate weight vectors, we express the observation z_*i*_(*t*) and desired states **w**_*i*_ (*t* + 1) with a discrete time Markovian state-space model:

(6) $$\begin{array}{c} {\text{z}}_{i}(t)=w_{i}{\left(t\right)}^{T}n(t)+s_{i}(t)\\ w_{i}(t+1)={\text{α w}}_{i}(t)+\lambda_{i}(t) \end{array}$$

where α is a scalar number that corresponds the weight vector at time *t* + 1 to the weight vector at time *t* with a one-order Markovian chain. We assume that the process noise term **λ**_*i*_ (*t*) of each channel has a normal distribution with zero average and covariance matrix **V**_i_ = *E*(**λ**_*i*_$\lambda_{i}^{T}$). In the observation model, we assume that the underlying clean intracortical signal *s_i_*(*t*) is a normal signal with zero mean and variance of *q* = *E*($s_{i}^{2}$). Based on the Kalman filter framework we can adaptively estimate our desired states **w**_i_ (t) in two steps:

(I) State update based on the previous state:

(7) $$\begin{array}{l} {\hat{w}}_{i}^{-}(t)={\text{α }\hat{w}}_{i}(t-1)\\ P_{i}^{-}(t)={\text{α }P}_{i}(t-1)+V_{i}(t) \end{array}$$

where $P_{i}^{-}$ (*t*) is the covariance matrix of estimation error considering the previous estimate of state **ŵ**_*i*_ (*t* − 1). The parameter α defining state-transition value can be identified in the simulation study in different conditions. Since, we have access to the true mixing weights in the simulation step, it is simply possible to identify alpha parameter by solving a least square problem. Assuming *X*_1_ = [*w_i_*(1) .... *w_i_*(*L* − 1) ] and *X*_2_ = [*w_i_*(2) .... *w_i_*(*L*) ], where *L* is the total number of sample points and *i* is the channel number. The parameter alpha corresponding to each channel can be identified as follows: α_*i*_ = *X*_2_$X_{1}^{T}$(*X*_1_*X*_1_^*T*^)^−1^.

(II) State modification based on the new measurement:

(8) $$\begin{array}{l} {\hat{w}}_{i}(t)={\hat{w}}_{i}^{-}\;(t)+K_{i}(t)\;[z_{i}(t)-n^{T}(t){\;\hat{w}}_{i}^{-}{}_{i}(t)]\\ P_{i}(t)=[I-K_{i}(t){\text{ n}}^{T}(t)]{\text{ P}}_{i}^{-}(t)\\ {\text{K}}_{i}(t)={\text{P}}_{i}^{-}(t)\text{ n}(t)\;{\left[n^{T}(t){\text{ P}}_{i}^{-}(t){\text{ n}}^{T}(t)+q\right]}^{-1} \end{array}$$

where **ŵ**_*i*_ (*t*) and **P**_*i*_ (*t*) are the updated state and updated covariance matrix of error after considering new measurement in computation, respectively. Kalman gain term, **K**_*i*_ (*t*) adjusts the contribution of new observation on the update of the state parameter. Kalman gain can be recursively updated according to the above equation.

### Data Collection

In this section, the simulation analysis is explained. First, we introduce the procedures for producing ground truth multichannel intracortical data. Then, we illustrate two types of simulated artifacts that can be commonly seen in BMIs during the freely moving condition. We also present different strategies for mixing true intracortical channels and synthetic artifacts. Afterward, the material and methods for recording physiological and behavioral data are introduced. We then explained the procedures for decoding force information form the real multichannel intracortical data.

#### Simulated Data

To evaluate the efficiency of the proposed spatial filtering algorithm in different conditions, we simulated clean multi-channel intracortical signals to obtain the ground truth signals. These ground truth brain signals can be combined with different types of artifacts in different simulated scenarios to investigate the efficiency of the proposed method. We produced simulated LFP channels using VERTEX toolbox in MATLAB to make sure that the ground truth intracortical channels are known (Tomsett et al., 2015). This toolbox simulates LFP signals recorded from large-scale neocortical tissues of macaque brain. In this toolbox, we specified one layer (same depth for all electrodes) in a cubic model of 16 electrodes. An inter-electrode spacing of 500 μm were specified for simulation similar to distance between wires of microwire array used for real data recording in this study. First, these settings produce 16 LFP channels with different temporal-spatial-spectral information. Second, lower frequencies contain higher power and represent 1/f falloff of the power like physiological data. Third, there is no artifact or noise in these simulated data and so we ensure that the original data is artifact-free. Hence, the VERTEX toolbox could provide us intracortical signals without any artifacts and as a result we could analyze the artifact removal algorithm in different conditions and scenarios. The clean simulated LFPs were generated at 1 KHz in 20 trials each with 20 s duration. We combined true multichannel signals **S** with simulated artifacts **N** (described in sections “Fast Outlier Artifacts” and “Motion Artifacts”) with a random mixing vector **γ**, sampled from −1 to 1 uniformly distributed random numbers:

(9) Z=S+β. γN

where **Z** shows multichannel mixed signals and β were defined to control the average input signal to noise ratio (SNR) of all channels in the analysis:

(10) $${\mathrm{SNR}}_{\mathrm{Input}}=10\log_{10}\frac{\left(E_{i}[\mathrm{var}(S)]\right)}{\left(E_{i}[\mathrm{var}(\text{β}.\;\mathrm{\gamma N})]\right)}$$

we employed coefficient of determination (*R*^2^) as the performance metric of removing artifacts from the noisy signals:

(11) $$R_{i}^{2}=1-\frac{\sum_{t=1}^{L}{\left(s_{t}-{\hat{s}}_{t}\right)}_{i}}{\sum_{t=1}^{L}{\left(s_{t}-\hat{s}\right)}_{i}}$$

where *s_t_*, *ŝ_t_*, and $\bar{s}$ are original signal, reconstructed signal and average of the original signal at sample point of *t*, respectively. Here *t* = 1, 2, ..., *L* with *L* total number of sample points. The average *R*^2^ of channels *i* = 1, 2, ..., *K* with *K* total number of channels, defines the performance of the artifact removal methods.

To evaluate the efficiency of the artifact removal method in non-stationary conditions, the random mixing vector **γ** are updated in various time steps (each 4, 2, and 0.5 s) by adding uniformly distributed random numbers (with standard deviations of 0.2) to the previous **γ** (Kelly et al., 2013). Hence, we change the spatial distribution of common artifact by introducing the random mixing vector **γ** and updates it temporally in specific time steps. We simulated two types commonly seen artifacts in the cortical recording of behaving subjects:

##### Fast outlier artifacts

**FIGURE 2:**
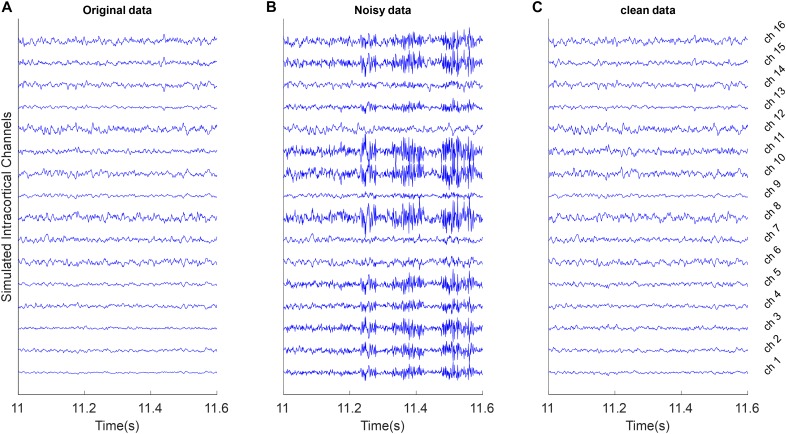
Example of removing outlier artifacts from the simulated multi-channel LFP data. All signals were shown with the same amplitude scale. **(A)** Simulated LFP channels without noise as ground truth in the simulation analysis. **(B)** The clean data were mixed with simulated random outlier artifacts distributed in channels with random scales and polarity. **(C)** Reconstructed data after removing artifacts from the noisy synthetic signals using the proposed weighed CAR algorithm.

To simulate these type of artifacts, it is assumed that **S** is true multichannel signals with *K* channels and *L* time samples. We computed the average vector μ and covariance of multichannel signal **C**_**S**_ and generate random patterns of outliers with 50 samples based on the K-dimensional normal distribution *N_K_*(**μ**, 5 **C**_**S**_). We selected $\frac{L}{50}$ of these random outlier artifacts without overlapping and combine them with true multichannel data based on the random mixing vector **γ**. Figure 2B shows an example of these artifacts.

##### Motion artifacts

**FIGURE 3:**
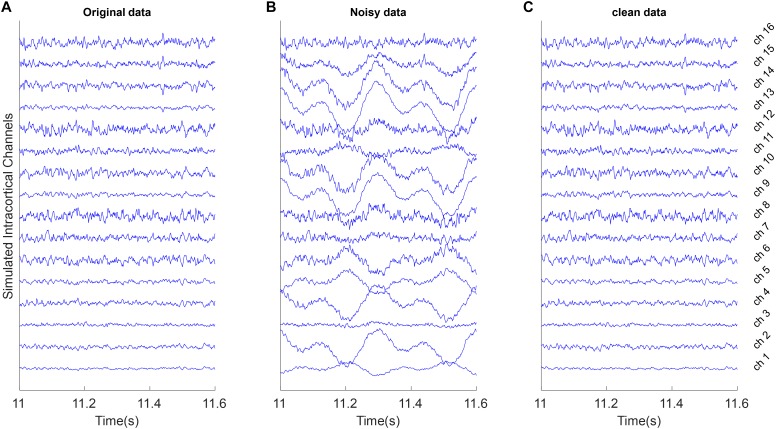
Example of removing motion artifacts from the simulated multi-channel LFP data. All signals were shown with the same amplitude scale. **(A)** Simulated LFP channels without noise as ground truth in the simulation analysis. **(B)** The clean data were mixed with the simulated random motion artifacts distributed in channels with random scales and polarity. **(C)** Reconstructed data after removing artifacts from the noisy synthetic signals using the proposed weighed CAR algorithm.

These types of artifacts often have slow variations with high amplitudes. We simulated motion artifacts by mixing 1.6 and 3.2 Hz sine functions (slow-varying oscillations) with true multichannel signals based on a random mixing vector **γ**. Figure 3B shows an example of these type of artifacts.

#### Real Data

All animal procedures were monitored and approved by the local ethics committee of animal care at Iran University of Science and Technology and were conducted in accordance with NIH protocols for animal research. Intracortical data were recorded from three Wistar rats (300–400 g) while the animals were pressing a force sensor located in front of them. Animals were trained to press a force sensor by their forepaw to receive a liquid reward. Force values were linearly converted to the deflection of a mechanical arm. Applying force to a predefined threshold would stop the mechanical arm in a position that animal could get a liquid reward. The animals were free to move in the experimental setup and there was no go cue or end cue to select beginning or end of each trial. In the Electrophysiology step, 16 channel micro wire arrays (4 × 4, 500 μm apart) were implanted in the forelimb region of primary motor cortex with stereotaxic coordinates +1.6 AP, −2.6 ML and −1.5 DV. Brain data were recorded at a rate of 10 KHz with multi-channel data acquisition system (Microprobes Inc., Gaithersburg, MD, United States). The force and brain signals were simultaneously recorded while animals performed the behavioral task. In the beginning of each session, a TTL pulse were sent to the recording systems to synchronize LFP and force signals. All the components of experimental setup including force sensor, mechanical arm and water pump were monitored or controlled using the Arduino micro controller based on a C++ script. The details of surgery and recording procedures have been previously published in (Khorasani et al., 2016).

##### Preprocessing

In the first step, different introduced artifact removal algorithms (CAR, median CAR, weighted CAR) were used to remove artifacts from the intracortical data recorded during force sensor pressing task. Before each method, intracortical channels were bandpass filtered (4th order Butterworth, band-pass filter, forward and backward) through 0.5–4,900 Hz to remove the DC offset and avoid the aliasing effect. Although the backward filtering cannot be used for the real-time applications, the causal and minimum phase version of this filter (forward filtering) produces phase lag equal to the order of the filter. Hence, considering 4th order Butterworth in real time, we will have 4 sample delay (4 ms considering 1 Khz sampling rate) that is completely acceptable for the real-time applications.

##### Feature extraction

Three type of features were extracted from the preprocessed data including LFP features, MUA features and LFP + MUA features. To obtain LFP features, the preprocessed multichannel brain signals were filtered through three spectral sub-bands (LF: 1–30 Hz), (MF: 30–120 Hz), and (HF: 120–300 Hz). Then, these bandpass filtered channels were rectified and lowpass filtered (4th order Butterworth, low-pass filter, forward and backward) to produce the envelope of different frequency bands. To extract MUA features, the high-frequency contents of data were obtained by applying a 300 Hz high-pass filter (4th order Butterworth, high-pass filter, forward and backward) on the preprocessed data. Then, the spike events were extracted by thresholding the band-pass filtered signal at 4 times the standard deviation of the amplitude of recording channel. The firing rates of these spike events were computed to create the MUA-based feature vector (Figure 1). In the LFP + MUA decoder, both LFP and MUA features were combined to create a combinational feature vector.

##### Force decoding model

In this step, the extracted features were normalized by subtracting the mean values and dividing by standard deviation of each feature (Khorasani et al., 2018). PLS regression model was used to model the relationship between the input feature vector and the output force signal (Figure 1). PLS method is a multivariate regression algorithm that iteratively decompose both input and output variables to a set of small number of components. Each component is identified in order to maximize the covariance between the input and output variables. The details of this method is described in Khorasani et al. (2016).

Decoding accuracy of force signal were computed by measuring the coefficient of determination (*R*^2^) between the actual and predicted force:

(12) $$R^{2}=1-\frac{\sum_{t=1}^{L}\left(f_{t}-{\hat{f}}_{t}\right)}{\sum_{t=1}^{L}\left(f_{t}-\bar{f}\right)}$$

where *f_t_*, $\hat{f}$ shows the real and predicted force value at sample point *t.* $\bar{f}$ defines the average of force signal on the test fold with *L* samples. *R*^2^ has a range between (−∞, 1) (Fagg et al., 2009). The *R*^2^ values were computed with fivefold cross validation method after shuffling the order of trials.

## Results

### Simulation Study Results

Figure 2A shows 600 ms of a 20 s simulated multichannel LFPs as the true underlying signal. Figure 2B shows the same section of the data when the true signal was polluted with outlier artifacts with average input SNR of −5 dB in condition that the random mixing vector was updated each 2 s. First, the simulated artifact has affected specific channels and with different power. Second, the simulated artifact presents very fast oscillation with standard deviation more than true signal. The goal of spatial filtering is removing artifacts from the noisy data to reconstruct the original data. Figure 2C visually depicts that by employing the proposed spatial filtering algorithm, the artifacts are well removed from the noisy data.

In Figure 3 motion artifacts with very low frequency oscillations were added to the true multichannel recording. As can be seen in Figure 3B some channels contain low frequency amplitudes that distributed differently on the true underlying channels. As it is shown in Figure 3C the weighted CAR algorithm removed these types of artifact.

**FIGURE 4:**
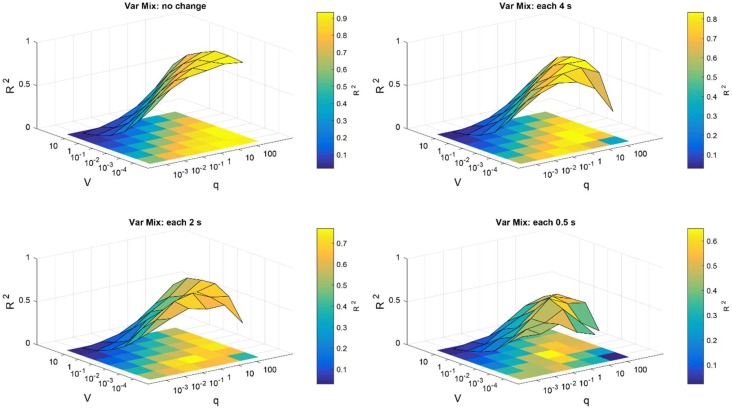
Parameter optimization of Kalman filter in the simulation study. Average *R*^2^ of intracortical channels between original and reconstructed signal were computed for different selections of noise process covariance matrix **V** and variance of observation model q. Parameters **V** and q were assumed constant for all intracortical channels. The parameter optimization was repeated for different simulation conditions described in the title of each figure.

To search for optimum parameters of weighted CAR filter that produce the highest and most stable *R*^2^, the average of all channels over 20 trials were computed for SNR input of −20 dB. The high *R*^2^ requires prefect match between the reconstructed and the original signal. Figure 4 presents the surface map of the average *R*^2^ between the original and reconstructed signal of all channels obtained from 20 trials for different scenarios. As it is shown, in case of slow changes of mixing matrix, the higher q and lower **V** lead to better higher *R*^2^ values. That means in this scenario, we can select wide range of values for the Kalman parameters. But, for the fast changes of mixing matrix we need to select more specific values to ensure the accuracy and stability of the artifact removal.

**FIGURE 5:**
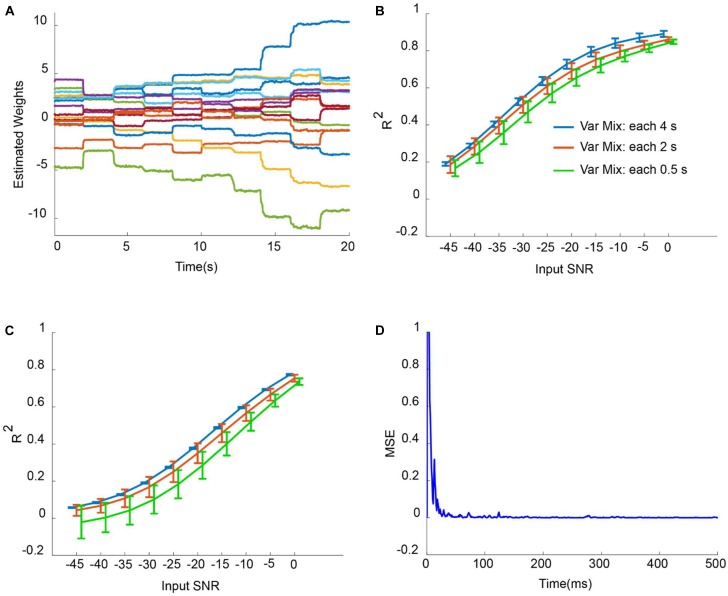
Artifact removal from simulated noisy data in different conditions. **(A)** Adaptation of weights in weighted CAR when the distribution of outlier artifact on the multichannel brain signals were updated each 2 s. **(B)** Average *R*^2^ for different input SNR and different mixing condition in case of adding outlier artifacts to true intracortical signals. **(C)** Average *R*^2^ for different input SNR and different mixing condition in case of adding motion artifacts to the true intracortical signals. **(D)** Example of average MSE of all channels for input SNR of –2 dB.

Figure 5A shows the adaptation of weights corresponding to weighted CAR filter *w_i_* (*t*) in situation that mixing vector has been updated each 2 s with input SNR of −2 dB. It is obvious that the proposed method has changed the weights of CAR filter to extract the true common artifact from the multichannel data. Figure 5B,C depict the average *R*^2^ of all intracortical channels for different input SNRs when mixing vector updated each 4, 2, and 0.5 s for outlier artifacts and motion artifacts, respectively. The average *R*^2^ was enhanced with increasing of input SNR for both outlier and motion artifacts. Moreover, fast changes of mixing vector resulted in less average *R*^2^ for different input SNRs. This can be explained because during the adaptation, it takes around 200 ms to find the true weights and so there are some difference between the reconstructed and original signal of all channels during this period. In the case of adding outlier artifact to the original intracortical channels, the proposed weighted CAR filter reconstructed the original signal with average *R*^2^ higher than 0.5 for input SNRs bigger than −25 dB. In the case of adding motion artifacts to the original intracortical channels, the proposed weighted CAR filter reconstructed the original signal with average *R*^2^ higher than 0.5 for input SNRs bigger than −10 dB. Figure 5D shows the mean square error (MSE) averaged over all channels in the case of the input SNR of −2 dB and update of mixing vector each 2 s. This figure shows the quick convergence of the estimation average error toward the steady-state error. We can simply identify parameter α in the simulation study in different conditions such as changing mixing vector each 4 and 2 and 0.5 s as described in the method section. The analysis finds α = 0.99 as a good selection in a condition that mixing vectors changes each 4 or 2 or 0.5 s. For the real applications, it is unlikely that the mixing vector changes faster than 0.5 s.

### Experimental Study Results

**FIGURE 6:**
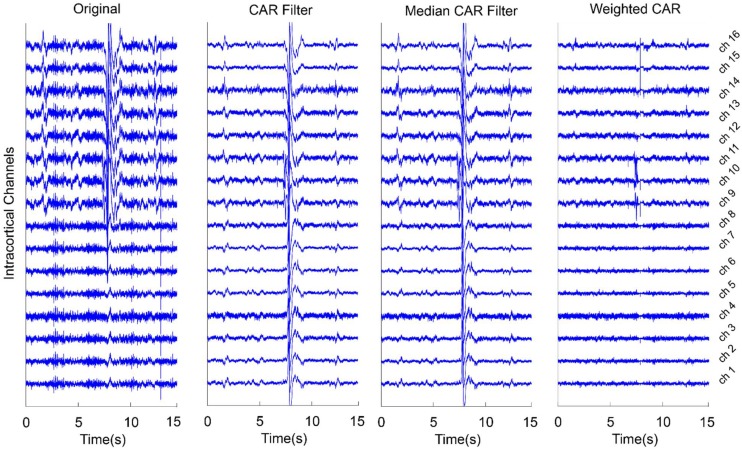
An example of using different CAR-based spatial filtering algorithm on removing artifacts from real intracortical signals of freely moving rats. In case of original data, no preprocessing was applied on the raw intracortical signals. In CAR Filter, common artifact was estimated using averaging of all channels. In median CAR, common artifact was estimated using median of all channels. In weighted CAR filter, the contribution of common artifact was adaptively adjusted based on the Kalman filter algorithm.

Figure 6 presents an example for the effect of different CAR-based spatial filtering on removing artifacts from the real intracortical channels. In the case of original data, no preprocessing was applied on the intracortical channels. It is obvious that the artifacts were distributed on the channels with different scales in some data points. CAR filter and median CAR filter could not efficiently remove these artifacts from the data. Visual inspection shows that weighted CAR more accurately removed these artifacts from the channels thanks to its Kalman filter adaptive structure.

**FIGURE 7:**
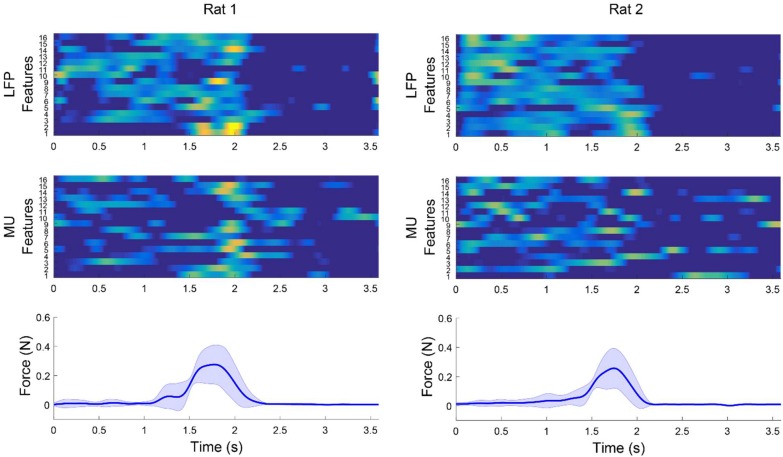
Example of modulation between multichannel LFP and MUA features and force values after applying weighted CAR filter averaged over 15 trails in each rat. Average LFP features were obtained by filtering preprocessed data between 120 and 300 Hz and computing envelope of each channel. Average MUA features were obtained by filtering preprocessed data in range of 3004,900 Hz and computing firing rate of cross-threshold voltages.

Figure 7 shows an example for modulation between multichannel LFP features and MUA features and force signal after applying weighted CAR filter. The average features obtained from 15 trials in each rat clearly demonstrate that the LFP power or MUA firing rate were increased during applying force on the force sensor and decreased by releasing the force sensor.

**FIGURE 8:**
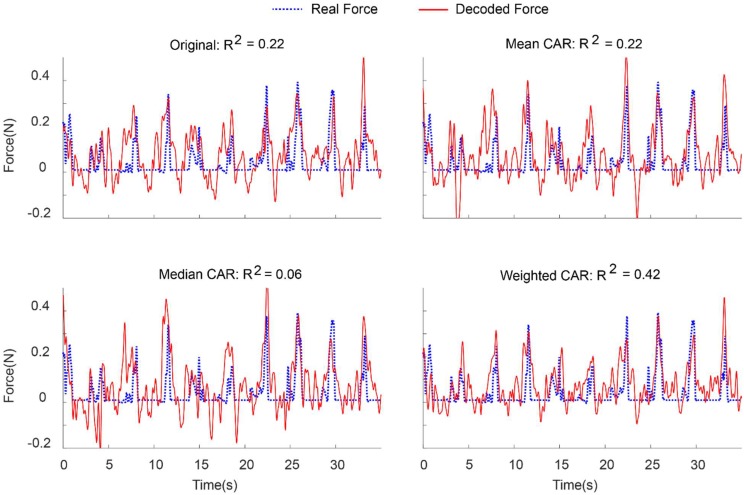
Representative example for decoding of continuous force signal from intracortical channels after applying different spatial filters.

Figure 8 demonstrates an example of force prediction from the combination of LFP and MUA features after applying different artifact removal algorithms. Applying weighted CAR algorithm resulted in precise decoding of force amplitude compared to original signal and standard CAR algorithms.

**FIGURE 9:**
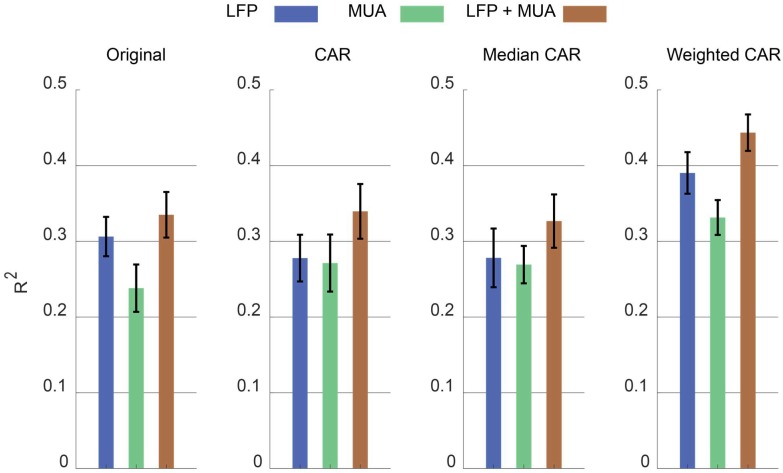
Comparison between decoding performance of the force signal using different input types (LFP, MUA, and LFP + MUA) and different spatial filtering algorithms (Mean CAR, Median CAR, and weighted CAR). Each bar shows mean ± standard error of *R*^2^ obtained from three animal datasets.

Figure 9 illustrates the error bar (mean ± standard error) of *R*^2^ value between the real and predicted force signal corresponding to different artifact removal algorithm. The aim is to find out which artifact removal algorithm produces significant improvement of decoding accuracy. The non-parametric Friedman test was applied on the obtained *R*^2^ values between predicted and real force signal and multiple comparison were performed using Tukeys honestly significant difference method (Daly et al., 2015). In the case of using both LFP and MUA for decoding, this analysis shows that weighted CAR significantly improves decoding performance compared with original case (*p* < 0.001), mean CAR (*p* < 0.01) and median CAR (*p* < 0.001) and other methods are not significantly different. In the case of using only LFP information for decoding, this analysis shows that weighted CAR significantly improves decoding performance compared with original case (*p* < 0.05), mean CAR (*p* < 0.001), and median CAR (*p* < 0.001) and other methods are not significantly different. In the case of using only MUA information for decoding, only weighted CAR significantly improves decoding performance compared with original case (*p* < 0.05) and other methods are not significantly different.

## Discussion

In this study, we presented an automatic CAR-based artifact removal algorithm for BMI applications. The algorithm automatically identifies the weight of common noise propagated on the channels sample by sample. All parameters of the method were kept fixed after setting the initial values. In the first step, it estimates the noise signal at each sample time by computing the average of all channels. We assume that the obtained noise signal is propagated through the channels with different amplitude and polarity. In the second step, the Kalman filter adaptively identify the amplitude and polarity of the common noise of each channel. The simulation study demonstrates that the weighted CAR quickly converges to the steady-state error in order to remove artifact from the noisy data. We evaluated the efficiency of the proposed method under different conditions. In the first condition, we assume that a noise source was distributed across the clean channels with random positive and negative scales. We showed that weighted CAR successfully reconstructs the original signal with positive *R*^2^ for input SNRs in range of −45 to 0 dB. We also evaluated a condition that the distribution of the noise source was changed at each 4, 2, and 0.5 s. The results showed that the proposed method can quickly identify the true weights of CAR filter even with quick changes of mixing vector at each 0.5 s for both outlier and motion artifacts. Moreover, offline analysis on the real intracortical data showed that the proposed adaptive weighted CAR filter improved decoding accuracy of continuous force information compared with non-adaptive mean CAR and median CAR algorithms.

Many algorithms have been proposed in the literature to remove artifacts from the brain data (Sweeney et al., 2013; Daly et al., 2015; Foodeh et al., 2017). In conditions that desired signal and noise do not have any spectral overlap, filtering-based algorithms can be used for removing artifacts (Sweeney et al., 2012). But, in the physiological recording, especially for broad-band brain data, such as LFPs and ECoGs, there are overlaps between spectral contents of the desired signal and noise. In this work, we applied the weighted CAR algorithm on the raw brain data, so the algorithm could remove the artifacts from the whole spectrum of data. Adaptive filtering algorithm is another solution to remove artifacts from the brain data. But these algorithms often need a reference signal to optimally filter out the artifact from the data. In many situations the reference is not available and adding reference may increase the complexity of the whole system for real-world BMI applications. In this study, we estimate the reference signal based on the CAR algorithm and use a Kalman algorithm to update the weights. Kalman filtering is more robust and more accurate in comparison to least mean square algorithms (Sweeney et al., 2012). One important advantage of this method is that it does not require any prior knowledge about the structure and the nature of the noise sources or the topography of the noise propagation on the brain channels. For example, independent component analysis (ICA) requires prior knowledge about the type and structure of the noise to separate the desired sources from the noisy sources (Zou et al., 2016). Furthermore, for optimum implementation of other spatial algorithms like Laplacian filters we need accurate knowledge about the spatial distribution of artifacts on the brain channels (Foodeh et al., 2017).

It should be noticed that CAR filter may remove the desired information from the recording channels. But the main point is that the CAR filter is a strong algorithm to improve the SNR in brain recording and this matter has been widely presented in the previous studies (Liu et al., 2015) and (Milekovic et al., 2015). That means there is a tradeoff between losing some information related to the state of behavior or action and removing the high level of noise. Hence, generally we will have better decoding performance since the level of noise in the common signal is much higher than the desired signal. The main problem of CAR filter is that in some situations we may have channel specific noises and so applying CAR filter propagates the channel specific artifacts through the clean channels. The obtained results in this study totally confirm this statement. First, the CAR filter has not significantly improved the decoding performance in comparison with the original signal supporting that CAR algorithm may make the condition worse. Second, the proposed CAR-based algorithm significantly improved the decoding performance in comparison with the original data proving that the CAR filter is a powerful algorithm, but it needs some modification for the efficient functionality.

Several methods have been proposed to extract more accurate estimation of common-mode artifacts. Liu et al. (2015) suggested a median CAR filter to spatially filter out the epidural field potentials because median values are less sensitive against outliers in the recording. But, using median CAR filter under normal condition may remove desired task-related information due to non-linear characteristics of median filtering (Rousseeuw and Hubert, 2011). Kelly et al. (2013) proposed an adaptive CAR filter to remove noises from the ECoG recording based on the combination of CAR algorithm with adaptive noise canceling filter. They showed that this method effectively outperforms conventional CAR filter on removing artifacts from ECoG data. But, the algorithm requires to be applied to sliding segment of the data that makes it less practical for real-time artifact rejection. Also, this method needs various optimizations to ensure convergence of the method. Erickson et al. (2016) proposed an iterative artifact removal algorithm by scaling noise template computed by median CAR in each identified noisy section of the data. This algorithm finds the corrupted window of brain channels and scales the common reference obtained from the smoothed median estimate of all channels.

We showed that the proposed artifact removal algorithm improves the accuracy of force signal decoding from LFPs better than MUAs. We showed that using the combination of LFPs and MUAs together with the proposed artifact removal algorithm lead to a high decoding performance. Moreover, the improvement of the decoding accuracy in case of using the LFP + MUA decoder over the LFP-only or the MUA-only decoders show that the LFPs and the MUAs may contain different or supplementary information about the covariate force signal.

The main reason for the poor performance during the stationary force (zero force) is that in the designed experimental setup, we could not continuously record the forelimb force. However, the results obviously show that we can distinguish force period from the non-force periods by applying a simple thresholding in the decoded force signal. For the real BMI applications, where the subject needs to continuously control an external device, there is no need to estimate force values at all times. For example, in the control of a neural prosthesis, we can decode the kinematic information such as the movement velocity or the limb position during the reaching phase and make the applied force zero and when we require the force information, the force-based decoder can control the prosthesis for object grasping or key pressing.

## Conclusion

We investigated the performance of adaptive weighted CAR for real-time artifact removal from intracortical channels. The proposed algorithm is automatic and does not need any knowledge about the content of brain recording. We analyzed the proposed method on both real and simulated intracortical data to test the power of the proposed method under different condition and scenarios. We showed that removing artifacts by using the proposed weighted CAR filter can significantly improve the decoding performance considering LFP or a mix of LFP and MUA as input compared with conventional CAR filters.

## Ethics Statement

This study was carried out in accordance with the recommendations of the responsible local ethical committee [Iran University of Science and Technology (IUST)]. The protocol was approved by the ‘IUST local committee of ethics.’

## Author Contributions

AK, VS, and MD designed the study, interpreted the data, wrote the initial draft of the paper, and revised and approved of the papers’ final version. AK performed the data analyses.

## Conflict of Interest Statement

The authors declare that the research was conducted in the absence of any commercial or financial relationships that could be construed as a potential conflict of interest.
